# Predicting Microbe-Disease Association by Learning Graph Representations and Rule-Based Inference on the Heterogeneous Network

**DOI:** 10.3389/fmicb.2020.00579

**Published:** 2020-04-15

**Authors:** Xiujuan Lei, Yueyue Wang

**Affiliations:** School of Computer Science, Shaanxi Normal University, Xi’an, China

**Keywords:** microbe-disease association, heterogeneous network, network embedding algorithm, Node2vec, skip-gram

## Abstract

More and more clinical observations have implied that microbes have great effects on human diseases. Understanding the relations between microbes and diseases are of profound significance for disease prevention and therapy. In this paper, we propose a predictive model based on the known microbe-disease associations to discover potential microbe-disease associations through integrating Learning Graph Representations and a modified Scoring mechanism on the Heterogeneous network (called LGRSH). Firstly, the similarity networks for microbe and disease are obtained based on the similarity of Gaussian interaction profile kernel. Then, we construct a heterogeneous network including these two similarity networks and microbe-disease associations’ network. After that, the embedding algorithm Node2vec is implemented to learn representations of nodes in the heterogeneous network. Finally, according to these low-dimensional vector representations, we calculate the relevance between each microbe and disease by utilizing a modified rule-based inference method. By comparison with three other methods including LRLSHMDA, KATZHMDA and BiRWHMDA, LGRSH performs better than others. Moreover, in case studies of asthma, Chronic Obstructive Pulmonary Disease and Inflammatory Bowel Disease, there are 8, 8, and 10 out of the top-10 discovered disease-related microbes were validated respectively, demonstrating that LGRSH performs well in predicting potential microbe-disease associations.

## Introduction

Varieties of microbial communities are dominant throughout the human different body niches including skin, mouth, respiratory tract, throat, stomach, gut and colon, which mainly compose of bacteria, protozoa, archaeon, viruses, and fungi (Methe et al., 2012; Althani et al., 2016). It is generally that a wide range of them play fundamental roles in human health and diseases such as maintaining homeostasis (Bouskra et al., 2008), developing the immune system (Round and Mazmanian, 2010; Gollwitzer et al., 2014) and resisting pathogens (Methe et al., 2012). For example, the majority of microbes reside in the gut, regulating human physiology and nutrition by modulating host metabolism and immunity. They can digest and convert dietary constituents into active forms (Qin et al., 2010; Ahn et al., 2013).

Microbial communities are considered as an essential “organ” governing health and disease, which can be influenced by host genetics and host environment such as feeding habits, life styles, seasons and antibiotics (Huttenhower et al., 2012; Althani et al., 2016). If the microbial communities become imbalanced, there may interfere with the symbiotic relationships and cause diseases. For instance, researchers found that the number of phylum Actinobacteria among diabetics was significantly lower than the healthy person (Long et al., 2017). In addition, some studies found a decrease in the relative percentage of Bacteroidetes in obese people compared to the general population (Ley et al., 2006). Moreover, low microbial diversity can lead to inflammatory bowel disease (IBD) (Qin et al., 2010). Thus, understanding the microbe-disease associations can help us know disease pathogenesis to boost disease diagnosis and therapy.

With the advances in sequencing technologies and bioinformatics, more and more microbes living in oceans, soil, human bodies and elsewhere began to be investigated by the scientific community (Gilbert and Dupont, 2011; Methe et al., 2012; Cenit et al., 2014). The Human Microbiome Project Consortium (HMP) was funded to explore the relationships between microbes and human diseases. It generates a wide range of quality-controlled resources and data to develop metagenomic protocols, which is available for scientific research (Methe et al., 2012). Ma et al. (2016) constructed The Human Microbe-Disease Association Database (HMDAD) through collecting correlations between microbes and diseases from 61 published literatures. These achievements provided the foundation for further research on using computational methods to predict potential associations.

In recent years, some computational methods have been conceived for predicting microbe-disease associations based on the assumption that similarly functioning microorganisms incline to share similar associations or non-associations with diseases. By using the Gaussian interaction profile (GIP) kernel similarity, Chen et al. (2017) developed a prediction method called KATZHMDA that infers potential associations based on the number and length of walks in a heterogeneous network. Li et al. (2019) constructed a bidirectional weighted network by combining a normalized Gaussian interaction scheme with a bidirectional recommendation model. Zou et al. (2017) used a bi-random walk and logistic function transformation on a heterogeneous network constructed based on the GIP kernel similarity. Through a combination of the GIP kernel similarity and LapRLS classification, Wang et al. (2017) designed a computing model LRLSHMDA, which is semi-supervised. Meanwhile, through integrating the GIP kernel similarity with disease symptom similarity, Qu et al. (2019) implemented the matrix decomposition and label propagation algorithm on the similarity network for associations’ prediction. Huang et al. (2017) predicted potential associations based on known microbe-disease bipartite graph and neighbor collaborative filtering. Moreover, Fan et al. (2019) proposed a method called MDPH_HMDA for prediction by executing standardized HeteSim measurements to weight the relations in a heterogeneous network combined by the GIP kernel similarity, the microbe–microbe functional similarity and the symptom-based human disease similarity. Niu et al. (2019) identified the potential associations by introducing the concept of hypergraph, which put all disease-related microbes on a single hyperedge. In order to take the unequal contributions of microbe and disease information into consider, Zhang et al. (2018) developed a bidirectional similarity integral label propagation method with calculating the microbe functional similarity and the disease semantic similarity.

At the same time, many network embedded methods have been proposed, such as DeepWalk (Perozzi et al., 2014), SDNE (Wang et al., 2016), Node2vec (Grover and Leskovec, 2016), etc. In this study, inspired by the performance of graph representations for many real-world problems such as protein network research, text and visual processing (Cao et al., 2016). We utilize Node2vec (Grover and Leskovec, 2016) to predict potentially unknown associations (LGRSH) on a heterogeneous network. First, similarity networks for microbes and diseases are calculated by the GIP kernel similarity. Then, we construct a heterogeneous network integrating the two similarity networks and known microbe-disease associations’ network. After that, the embedding algorithm Node2vec has been utilized to assign a low-dimensional vector representation to nodes in the heterogeneous network. Finally, according to the vector representation of each node, we calculate the degrees of correlation between microbes and diseases to discover potential associations with a modified rule-based inference method. In order to assess the prediction performance of LGRSH, we implemented Leave-one-out cross validation (LOOCV) and fivefold cross validation. The area under the receiver operating characteristic curve (AUC) obtained by LGRSH are 0.9260 and 0.9254, which is better than the compared methods. Moreover, case studies of asthma, Chronic Obstructive Pulmonary Disease (COPD) and IBD demonstrate that LGRSH can be considered as an effective method for association prediction.

## Materials and Methods

### Material

We download microbe-disease associations from HMDAD (Ma et al., 2016), which contains 483 verified associations’ records between 292 microbes and 39 diseases. After removing the repetitive relationships, 450 distinct associations’ records are obtained. Then we construct a 39 × 292 dimensional adjacency matrix *MD* of the associations’ network. *MD* (*i*, *j*) is 1 indicating that there is a known association between disease *d*(*i*) and microbe *m*(*j*), otherwise, *MD* (*i*, *j*) is 0.

### Methods

**FIGURE 1:**
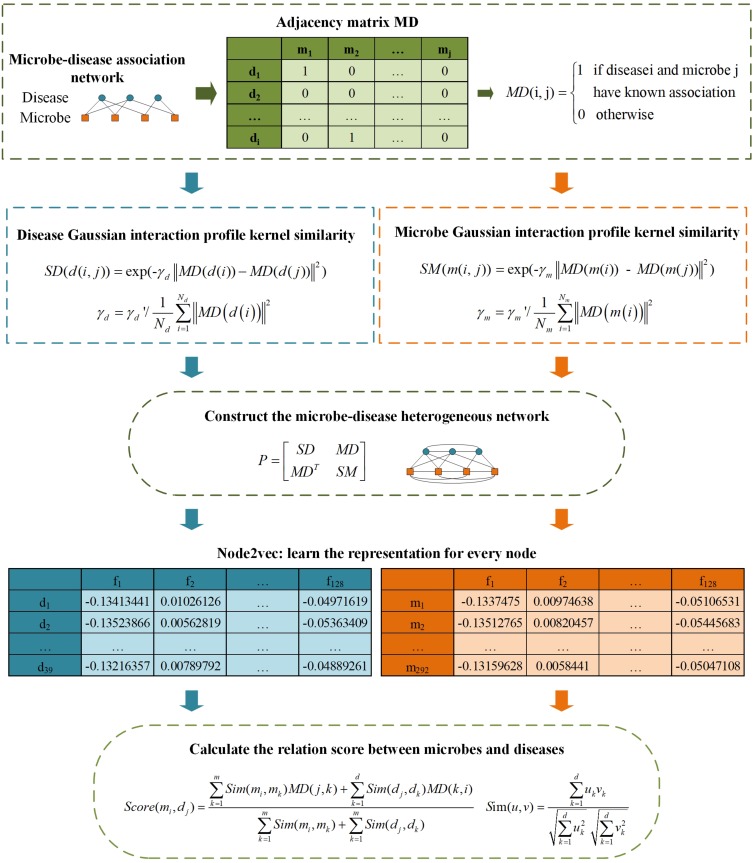
The flowchart of LGRSH.

As illustrated in Figure 1, firstly, the similarity networks for microbe and disease have been constructed. And then, a heterogeneous network integrating two similarity networks and microbe-disease associations’ network can be obtained. After that, the embedding algorithm Node2vec is utilized to learn the representation for every node. Finally, according to the topology information based on Node2vec method, we calculate the relation score between every microbe vector and disease vector.

#### Calculation of Microbe Similarities Based on the GIP Kernel Similarity

Based on the assumption that two microbes are more likely to share functional similarities potentially if they are related to more common diseases. We calculate the GIP kernel similarity for microbes based on known microbe-disease associations’ network. For microbes *m(i)* and *m(j)*, the similarity score is obtained according to Eq. (1) (Wang et al., 2017):

(1) $$S\,M\,\left(m\,\left(i,\,j\right)\right)=\exp\left(-\gamma_{m}\,{||M\,D\,\left(m\,\left(i\right)\right)-M\,D\,\left(m\,\left(j\right)\right)||}^{2}\right)$$

where *m(i, j)* represents two arbitrary microbes in matrix *MD*. Parameter γ*_*m*_* is used to control the bandwidth and is affected by a new bandwidth parameter γ*_*m*_’*(Wang et al., 2017), which can be obtained as Eq. (2):

(2) $$\gamma_{m}=\gamma_{m}^{\prime }/\frac{1}{N_{m}}\,\sum_{i=1}^{N_{m}}{||M\,D\,\left(m\,\left(i\right)\right)||}^{2}$$

here, *N*_*m*_ is equal to 292, which indicates the total number of microbes. The parameter γ*_*m*_’* is set to 1 for simplicity (Wang et al., 2017).

#### Calculation of Disease Similarities Based on the GIP Kernel Similarity

In the similar way, we construct a disease similarity network by using the GIP kernel similarity for each disease pair. The similarity between disease *d(i)* and *d(j)* is obtained according to Eq. (3) (Wang et al., 2017):

(3) $$S\,D\,\left(d\,\left(i,j\right)\right)=\exp\left(-\gamma_{d}\,{||M\,D\,\left(d\,\left(i\right)\right)-M\,D\,\left(d\,\left(j\right)\right)||}^{2}\right)$$

where *d(i, j)* represents two arbitrary diseases in matrix *MD*. The parameter γ*_*d*_* can be obtained as Eq. (4):

(4) $$\gamma_{d}=\gamma_{d}^{\prime }/\frac{1}{N_{d}}\,\sum_{i=1}^{N_{d}}{||M\,D\,\left(d\,\left(i\right)\right)||}^{2}$$

here, *N*_*d*_ is equal to 39, which indicates the total number of diseases. The parameter γ*_*d*_’* is set to 1 for simplicity (Wang et al., 2017).

#### Constructing a Heterogeneous Network for Microbes and Diseases

According to the Eqs (1) and (3), we have constructed two similarity matrices SM and SD. Then we construct a heterogeneous network including the edges of microbe–microbe, microbe-disease and disease–disease associations, and it can be expressed as Eq. (5):

(5) $$P=\left[\begin{array}{cc} S\,D & M\,D\\ M\,D^{T} & S\,M \end{array}\right]$$

where *P* represents the matrix of heterogeneous network. *MD*^*T*^ is the transpose of *MD*.

#### Using Node2vec to Learning Representations

Node2vec is a flexible neighborhood sampling strategy which can explore neighborhoods in the form of Breadth-First Sampling (*BFS*) and Depth-First Sampling (*DFS*) fashion by introducing two parameters (Grover and Leskovec, 2016). It maximizes the network neighborhood of nodes by mapping nodes to vector feature spaces. Therefore, we apply Node2vec to learn vector representations for nodes in the heterogeneous network.

**FIGURE 2:**
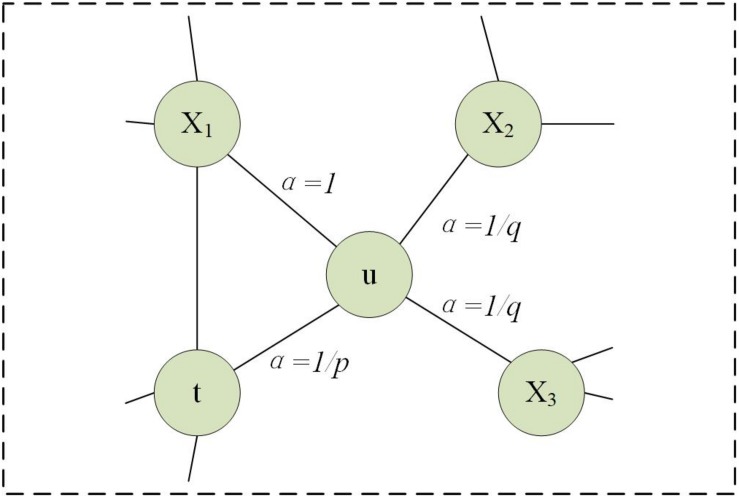
Description of walking strategy in Node2vec when the traversal has just gone from *t* to *u*.

Firstly, we utilize a bias random walk strategy to calculate the transition probabilities for every node. For a current node *u*, the probability of accessing the next node *x* can be calculated as follows:

(6) $$P(c_{i}=x|c_{i-1}=u)=\{{}_{0}^{\frac{{\text{π}}_{ux}}{Z}}{\;}_{otherwise}^{if\left(u,x\right)\;\in \;E}$$

here, *Z* is a regularization constant. π*_*ux*_* is denormalized transition probabilities on edges (*u*, *x*) leading from *u*, which is influenced by a weight adjustment parameter α. We suppose the walk just went from *t* to *u* and setπ_*ux*_ = α_*pq*_(*t*, *x*)⋅*w*_*ux*_, where

(7) $$a_{p\,q}\,\left(t,x\right)=\left\{ \begin{array}{c} 1\,/\,p\;i\,f\,d_{t\,x}=0\\ 1\;i\,f\,d_{t\,x}=1\\ 1\,/\,q\;i\,f\,d_{t\,x}=2 \end{array}\right.$$

here, *d*_*tx*_ is in the range of {0, 1, 2}, representing the shortest distance from nodes *t* to *x*. Parameters *p* and *q* are used to strike a balance between *DFS* and *BFS*. As shown in Figure 2, parameter *p* is a return parameter that affects the possibility of re-traversing a node immediately during a walk. If *p* is set to be larger, it is less likely to revisit the node that was just accessed. This strategy can lead to moderate exploration and avoid repetitive sampling. If the value is set to be smaller, the walk is more likely to backtrack, and tends to reach nodes near the node. There is more concerned for the local information. Parameter *q* is an in-out parameter, which allows searches to distinguish “inward” and “outward” nodes (Zeng et al., 2019). If *q* > 1, the walk tends to be closer to node *u*. In contrast, if *q* < 1, it tends to traverse nodes far from node *u* (Zeng et al., 2019).

We first select one node *u* and mark it as the current node, and then select one node *v* from all the neighbors of the current node *u* based on the transition probabilities calculated above. Following, we mark this newly selected node *v* as the current node and repetitive such as a node sampling process. The algorithm terminates when the number of nodes in a sequence reaches a preset walking length *l*. By referring to the previous paper, we set *l* as10 (Munui et al., 2018).

**FIGURE 3:**
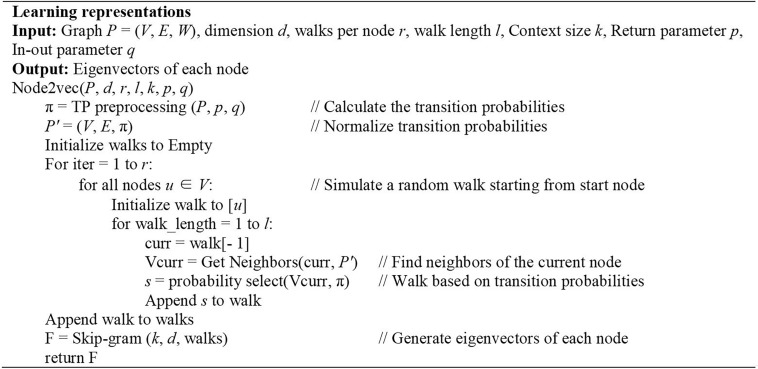
Description of algorithm Node2vec.

Node2vec uses Skip-gram model to generate eigenvectors of nodes (Jang et al., 2019). Skip-gram model is a word embedding algorithms for learning distributed vector representations from a large number of textual corpora which tries to categorize a word according to other words in the same sentence as much as possible (Mikolov et al., 2013). In fact, the sequence of nodes obtained by bias random walk algorithm, each node actually corresponds to a word. The input of this model is the sequence encoding of a node, and the output is the nodes before and after the sequence. In this paper, we set the context size to 10 and the dimension of these eigenvectors to 128 according to the original parameter selection for the best performance (Grover and Leskovec, 2016). The algorithm is detailed in Figure 3.

#### Association Discovering

According to the popular rule-based inference method for predicting novel drug-target associations based on indirect relationships in 2017 (Zong et al., 2017), we utilize a modified Scoring mechanism to grade microbe-disease relations based on the low-dimensional vector representation. Considering that indirect relationships do not fully predict the relationship if there are few known relations between some microbes and diseases, especially if there is only single relationship, we have used both direct and indirect connections to calculate correlations between microbes and diseases.

We use *Score(m_*i*_, d_*j*_)* to represent the correlation score between the *ith* microbe and *jth* disease in the heterogeneous network. It can be calculated according to Eq. (8):

(8) $$S\,c\,o\,r\,e\,\left(m_{i},d_{j}\right)=\frac{\sum_{k=1}^{m}S\,i\,m\,\left(m_{i},m_{k}\right)\,M\,D\,\left(j,k\right)+\sum_{k=1}^{d}S\,i\,m\,\left(d_{j},d_{k}\right)\,M\,D\,\left(k,i\right)}{\sum_{k=1}^{m}S\,i\,m\,\left(m_{i},m_{k}\right)+\sum_{k=1}^{m}S\,i\,m\,\left(d_{j},d_{k}\right)}$$

In this Equation, *m* and *d* indicate the numbers of microbe and disease, *MD(i, j)* is the association between disease *i* and microbe *j*. The *Sim(u, v)* is calculated as Eq. (9):

(9) $$S\,\text{im}\,\left(u,v\right)=\frac{\sum_{k=1}^{d}u_{k}\,v_{k}}{\sqrt{\sum_{k=1}^{d}u_{k}^{2}}\,\sqrt{\sum_{k=1}^{d}v_{k}^{2}}}$$

here, *d* represents the dimension for each vector, *u*_*k*_, *v*_*k*_ represent the components of vectors *u* and *v*.

## Results

We implement LOOCV and fivefold cross validation on HMDAD to assess the prediction performance of LGRSH. In the LOOCV, we regard each known association as a test sample, with other known associations as training samples (Quan et al., 2014). All unverified microbe-disease associations are regarded as candidate samples. In the fivefold cross validation, we randomly divide all known microbe-disease associations into 5 average groups. Each of these five groups is regarded as testing sample, while other four groups are training samples. This process is conducted five times to mitigate the bias due to random sample partitioning (Niu et al., 2019). Based on the prediction score, we evaluate the predictive performance by ranking the test samples. The AUC can be calculated according to the receiver operating characteristic (ROC) curve. If there is a random prediction performance, the AUC value is 0.5.

### Effect of Parameters

**FIGURE 4:**
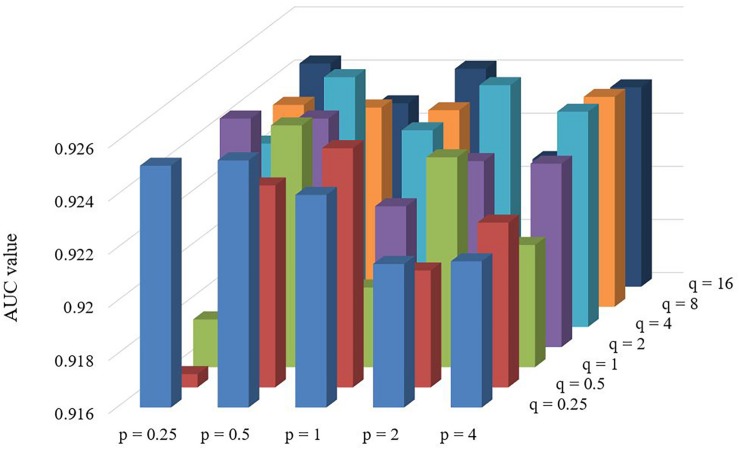
Effect of parameters *p* and *q* in fivefold cross validation.

There are two important parameters in Node2vec. One is a return parameter *p* and another is an in-out parameter *q*. We set various values under the framework of fivefold cross validation in order to evaluate the impact of these parameters. According to the comparison results in Table 1 and Figure 4, we can find that the performance of LGRSH is best with 0.9254 while *p* = 0.5, *q* = 4. Hence, we set *p* = 0.5, *q* = 4 in the subsequent experiments.

**TABLE 1:** Effect of parameters *p* and *q* in fivefold cross validation.

	*q* = 0.25	*q* = 0.5	*q* = 1	*q* = 2	*q* = 4	*q* = 8	*q* = 16
*p* = 0.25	0.9251	0.9165	0.9178	0.9246	0.9229	0.9236	0.9244
*p* = 0.5	0.9253	0.9236	0.9251	0.9246	**0.9254**	0.9235	0.9229
*p* = 1	0.9240	0.9250	0.9190	0.9213	0.9234	0.9234	0.9242
*p* = 2	0.9214	0.9204	0.9239	0.9230	0.9251	0.9181	0.9208
*p* = 4	0.9215	0.9222	0.9206	0.9229	0.9241	0.9239	0.9235

*Bold values: LGRSH achieves the best performance while *p* = 0.5, *q* = 4.*

### Comparison With Other Methods

**FIGURE 5:**
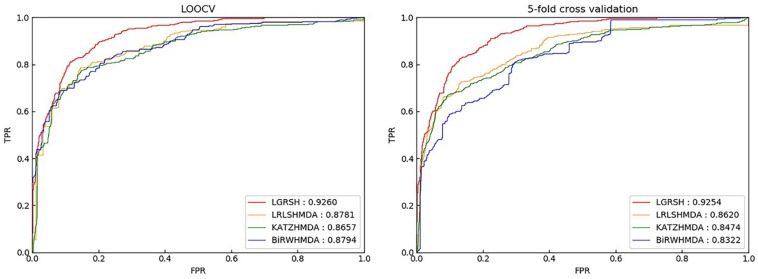
Prediction comparison between LGRSH and other three methods in LOOCV and fivefold cross validation while *p* = 0.5, *q* = 4.

**FIGURE 6:**
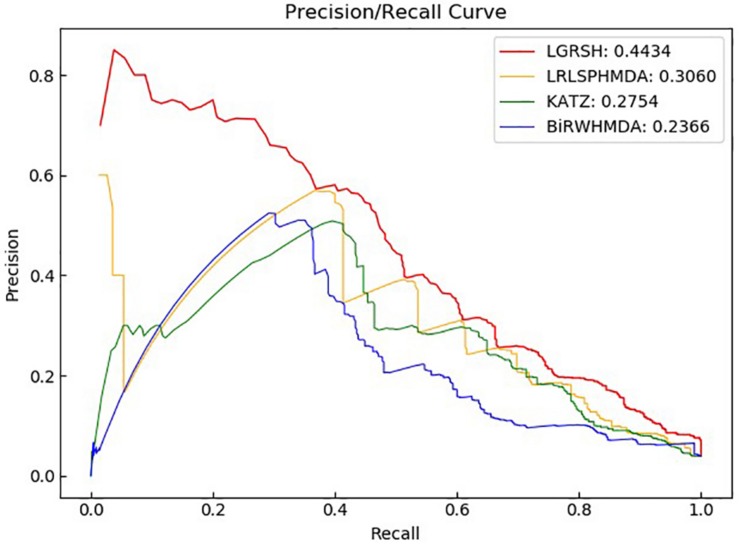
Precision-recall curves for LGRSH and other three methods in fivefold cross validation.

We compare LGRSH with three methods including LRLSHMDA (Wang et al., 2017), KATZHMDA (Chen et al., 2017) and BiRWHMDA (Zou et al., 2017). These four methods are measured by Precision-recall curve. As illustrated in Figures 5, 6, we can draw a conclusion that LGRSH performs better than other three methods.

**FIGURE 7:**
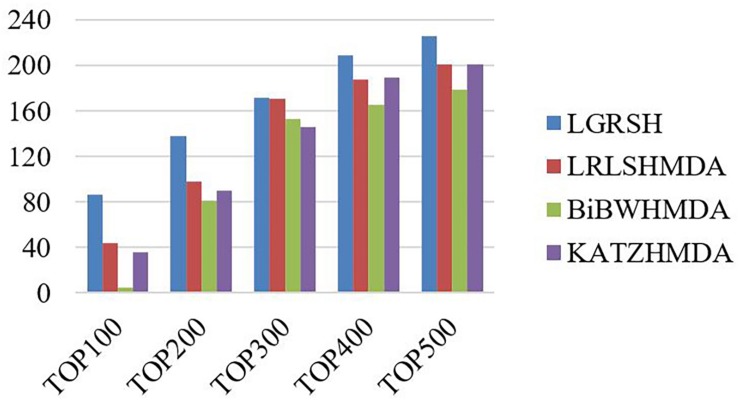
The number of correctly predicted by LGRSH and other three methods on HMDAD.

Furthermore, we measure the top-level results of LGRSH and three other methods in LOOCV. As shown in Figure 7, LGRSH can find more known associations among the top 500 predicted microbes.

## Case Studies

To evaluate the ability of LGRSH for discovering unknown associations in HMDAD, we implement case studies in asthma, COPD and IBD. We conduct experiments for 10 times on each diseases to make the results more stable. After calculating the similarity of every microbe and disease, the scores are sorted in descending order to obtain the top-10 candidate microbes for every disease. The scores of top-10 disease-related microbes are provided in Supplementary Tables S1–S3, respectively.

### Asthma

Asthma is a common inflammatory disease affecting more than 300 million people all over the world, which is more common in childhood with recurrent cough, wheezing and breathing difficulties. In recent years, asthma has been found to be closely linked with microbes (Caliskan et al., 2013). Hence, we consider Asthma for case studies. As shown in Table 2, 8 of top-10 discovered microbes were confirmed. For instance, Clostridium difficile colonization (ranked 1st in the list) in 1 month was associated with asthma between the ages of 6 and 7 (van Nimwegen et al., 2011). Researchers also proved that colonization with Clostridium coccoides (ranked 3rd in the list) and Bacteroides (ranked 7th in the list) at 3 weeks were associated with positive predictors of asthma at age 3 (Carl et al., 2008, 2011). In addition, the abundance of Firmicutes (ranked 2nd in the list) and Enterobacteriaceae (ranked 5th in the list) were higher in severe asthmatics compared with non-asthmatic people, while Actinobacteria (ranked 4th in the list) and Lachnospiraceae (ranked 9th in the list) with lower proportion (Marri et al., 2013; Ciaccio et al., 2015; Zhang et al., 2016; Li et al., 2017). Moreover, Huang et al. (2018) found that Lactobacillus (ranked 6th in the list) can reduce asthma severity and improve asthma control, which is beneficial to children with asthma.

**TABLE 2:** Validation results for Top-10 predicted microbes related with asthma.

Rank	Microbe	Evidence
1	Clostridium difficile	PMID:21872915
2	Firmicutes	PMID:27078029
3	Clostridium coccoides	PMID:21477358
4	Actinobacteria	PMID:30286807
5	Enterobacteriaceae	PMID:28947029
6	Lactobacillus	PMID:30400588
7	Bacteroides	PMID:18822123
		PMID:29161087
8	Burkholderia	**Unconfirmed**
9	Lachnospiraceae	PMID:28912020
10	Enterococcus	**Unconfirmed**

### Chronic obstructive pulmonary disease (COPD)

Chronic obstructive pulmonary disease is a progressive obstructive pulmonary disease with main symptoms of breathing difficultly and coughing (Rabe et al., 2007). It is more common among smokers, and is also influenced by factors like air pollution and genetics. Although the disease can be slowed down by treatment, there is still no clear treatment or pathogenesis for it. Recently, some findings indicate that changes in microbes may have significant effects in the development of COPD (Malhotra and Henric, 2015). Thus, we consider COPD for case studies. As shown in Table 3, 8 of top 10 discovered microbes were confirmed. For example, the main flora of Proteobacteria (ranked 1st in the list) and Bacteroidetes (ranked 5th in the list) increased with the deterioration of COPD (Rohde et al., 2004). Researchers also found that Helicobacter pylori (ranked 3rd in the list) infection is associated with reduced lung function and systemic inflammation in COPD patients (Mammen and Sethi, 2016). In patients with COPD, the proportion of Prevotella (ranked 2nd in the list) is reduced compared with healthy people, but phyla Actinobacteria (ranked 4th in the list), Clostridium difficile (ranked 6th in the list) and Lactobacillus (ranked 8th in the list) are increased (Yadava et al., 2016; Larsen, 2017; de Miguel-Diez et al., 2018; Ghebre et al., 2018). For example, the Clostridium difficile is twice as high in COPD patients as in healthy person. Moreover, Staphylococcus aureus (ranked 10th in the list) has been found in the respiratory tract of patients with COPD (Uddin et al., 2019).

**TABLE 3:** Validation results for Top-10 predicted microbes related with COPD.

Rank	Microbe	Evidence
1	Proteobacteria	PMID:29579057
2	Prevotella	PMID:28542929
3	Helicobacter pylori	PMID:28558695
4	Actinobacteria	PMID:29709671
5	Bacteroidetes	PMID:29579057
6	Clostridium difficile	PMID:30430993
7	Clostridium coccoides	**Unconfirmed**
8	Lactobacillus	PMID:26630356
9	Lachnospiraceae	**Unconfirmed**
10	Staphylococcus aureus	PMID:30804927

### Inflammatory bowel disease (IBD)

Inflammatory bowel disease is a chronic, idiopathic gastrointestinal inflammatory disease that is thought to be influenced by environmental and host factors (D’Aoust et al., 2017). It is characterized by recurrent episodes, diverse clinical manifestations and severe complications such as bleeding, abscess formation and perforation (Cosnes et al., 2002). In this paper, we consider IBD for case studies. As shown in Table 4, 10 of top-10 discovered microbes were confirmed. For instance, researchers have found that IBD is related to gut microbiological disorders including expansion of Enterobacteriaceae facultative anaerobic bacteria (ranked 8th in the list) and decrease in some beneficial fecal bacteria such as Firmicutes (ranked 5th in the list) (Eom et al., 2018; Zuo and Ng, 2018). In patients with IBD, the dominant of Prevotella (ranked 1st in the list), Veillonella (ranked 9th in the list) and Haemophilus (ranked 10th in the list) were largely contribute to dysbiosis (Said et al., 2014). Bacteroidetes (ranked second in the list) and Lactobacillus (ranked 7th in the list) were significantly increased compared with healthy people, but the Clostridium coccoides (ranked 6th in the list) was less abundant (Sokol et al., 2009; Thomas et al., 2015; Eom et al., 2018). Researchers also found that Clostridium difficile (ranked 3rd in the list) infection has become a significant clinical challenge for patients suffering from IBD, which can worsen flares of IBD, inducing to emergent colectomies and mortality (Hashash and Binion, 2014). Moreover, recent experimental results found that chronic infection with Helicobacter pylori (ranked 4th in the list) is protective against IBD. And IBD patients are least likely to be infected with Helicobacter pylori compared to the normal population (Sonnenberg and Genta, 2012; Kyburz and Muller, 2017).

**TABLE 4:** Validation results for Top-10 predicted microbes related with IBD.

Rank	Microbe	Evidence
1	Prevotella	PMID:24013298
2	Bacteroidetes	PMID:29492876
3	Clostridium difficile	PMID:24838421
4	Helicobacter pylori	PMID:22221289
		PMID:28124160
5	Firmicutes	PMID:25307765
		PMID:29492876
6	Clostridium coccoides	PMID:19235886
7	Lactobacillus	PMID:26340825
8	Enterobacteriaceae	PMID:30319571
9	Veillonella	PMID:30573380
10	Haemophilus	PMID:24013298

## Conclusion

There are countless microbe communities inhabited in the human body, having important impacts on human health and disease by regulating the metabolism and immunity. With the establishment of relational databases for microbes and diseases, exploring their associations have become a hot topic for researchers. In this study, we propose a predictive approach called LGRSH by utilizing network embedding algorithm Node2vec to obtain the representation for every node in the heterogeneous network. According to the vector representation for every node, we rank the relevance of each microbe vector and disease vector to discover potential microbe-disease associations. In LOOCV and 5-fold cross validation, LGRSH performs better compared with three other methods with AUC reached 0.9260 and 0.9254. The case studies of asthma, COPD and IBD show that LGRSH can be used as a predictive tool for microbe-disease associations.

Certainly, there are still some deficiencies in LGRSH. For example, there are only 450 know micro-disease associations, which accounts for very small proportion of human microbial diseases. This may result in less comprehensive for prediction. We believe that the problem will be solved when more microbe-disease links are discovered. In addition, the embedding algorithm itself is a local method. In the future, we will learn more graph representation algorithms to improve the global capability. Moreover, we calculate the similarities for microbe and disease through the GIP kernel, which may biased toward microbes and diseases with more known associations. Hence, we will improve the efficiency of LGRSH by integrating some optimization strategies such as microbe functional similarity, disease semantic similarity and symptom-based disease similarity in the future work.

## Data Availability Statement

The raw data supporting the conclusions of this manuscript will be made available by the authors, without undue reservation, to any qualified researcher.

## Author Contributions

XL and YW conceptualized the study and read and approved the final manuscript. YW conducted the experiments, analyzed the result, and wrote the manuscript. XL conceived the project, analyzed the result, and revised the manuscript.

## Conflict of Interest

The authors declare that the research was conducted in the absence of any commercial or financial relationships that could be construed as a potential conflict of interest.
